# Positive Association between Hemoglobin Concentration and Blood Pressure in Adults: A Cross-Sectional Study Based on Rafsanjan Cohort Study

**DOI:** 10.1155/2023/6283711

**Published:** 2023-02-01

**Authors:** Gholamreza Bazmandegan, Mitra Abbasifard, Hamid Ostadebrahimi, Mohammadreza Gholamrezapour, Zahra Kamiab

**Affiliations:** ^1^Non-Communicable Diseases Research Center, Rafsanjan University of Medical Sciences, Rafsanjan, Iran; ^2^Department of Physiology and Pharmacology, School of Medicine, Rafsanjan University of Medical Sciences, Rafsanjan, Iran; ^3^Department of Internal Medicine, Ali-Ibn Abi-Talib Hospital, School of Medicine, Rafsanjan University of Medical Sciences, Rafsanjan, Iran; ^4^Department of Pediatrics, Ali-Ibn Abi-Talib Hospital, School of Medicine, Rafsanjan University of Medical Sciences, Rafsanjan, Iran; ^5^Clinical Research Development Unit, Ali-Ibn Abi-Talib Hospital, Rafsanjan University of Medical Sciences, Rafsanjan, Iran; ^6^Department of Community Medicine, School of Medicine, Rafsanjan University of Medical Sciences, Rafsanjan, Iran

## Abstract

**Introduction:**

Identification of factors associated with blood pressure (BP), including hemoglobin, can be used in diagnosing, controlling, and predicting the prognosis of patients. This study aims to investigate the cross-sectional association between hemoglobin concentration and BP in people aged 35–70 years in a cohort study of Rafsanjan, Iran.

**Method:**

This cross-sectional study was conducted on 9398 urban and rural population of Rafsanjan adult cohort study as a part of the prospective epidemiological research studies in Iran (PERSIAN). Demographic information, medical history, history of smoking and alcohol intake, systolic and diastolic BP, and hemoglobin concentration were collected. A logistic regression test was performed to evaluate the relationship between hemoglobin concentration and BP in 4 unadjusted and adjusted models based on demographic indicators, clinical and laboratory findings using SPSS.24 software and SAS software version 9.2.

**Results:**

The mean age of the participants was 49.78 ± 9.53 years, and 53.2% (5002 people) were women. Adjusted models 3 and 4 showed a positive association between BP and hemoglobin. For each unit increase in hemoglobin, the odds ratio (OR) of BP in the adjusted model 3 was 1.062 (95% CI: 1.005–1.121), and in the adjusted model 4, it was 1.090 (95% CI: 1.031–1.153).

**Conclusion:**

Based on the results, the positive trend of BP and hemoglobin levels may indicate the need to pay more attention to these people as higher-risk groups for hypertension.

## 1. Introduction

Blood pressure (BP), as one of the vital signs, is expressed as the systolic pressure over the diastolic pressure. Systolic BP is caused when the heart muscle contracts, and diastolic BP is caused in the arteries when the heart returns to its original volume after contraction [1, 2]. According to the American College of Cardiology/American Heart Association, systolic BP less than 120 mm·Hg and diastolic BP less than 80 mm Hg are defined as normal for adults. Hypertension (HTN) is also a complication in which BP is chronically higher than 120/80 mm·Hg [3]. HTN is one of the most important causes of cardiovascular disease (CVD), stroke, and kidney failure in adults. So that, 51% of deaths due to stroke and 45% of deaths due to CVD are caused by HTN [4, 5]. The global prevalence of HTN is about one billion people, and approximately 7.1 million annual deaths can be attributed to HTN [6, 7]. The prevalence of this disease is increasing in all countries, especially Iran [8, 9]. According to the Ministry of Health and Medical Education in Iran, 20 out of every 100 adults have HTN [10].

Studies have reported several risk factors for HTN. The prevalence of the disease is different in males and females. The risk of developing HTN increases with age. Smoking and especially cigarette smoking, family history of HTN, obesity and overweight, and hyperlipidemia are also among the factors associated with the disease [11–13]. In a study on the factors affecting the incidence of HTN in people aged over 35 years using the logistic regression model, variables, including age, gender (male more than female), smoking, family history of HTN, obesity, and high triglycerides were the risk factors associated with HTN [14]. In general, the factors associated with HTN can be classified into three categories, including unchangeable factors (age, gender, and premature menopause), changeable factors (stress, obesity, diabetes, insufficient physical activity, smoking, and high low-density lipoprotein (LDL)), and unconfirmed risk factors (insufficient intake of B vitamins and hyperinsulinemia) [15].

Some studies have shown that hemoglobin levels are associated with HTN. Systolic and diastolic BP may increase by rising hemoglobin. High hemoglobin can cause blood vessels contraction, and then increases blood pressure. However, some other studies have reported that BP is not associated with blood viscosity in healthy individuals [16, 17]. The results of the study by Atsma et al. on healthy blood donors in the Netherlands showed that hemoglobin levels were positively correlated with BP [17]. In another study, Xuan et al. reported that there was a positive relationship between hemoglobin levels and systolic and diastolic BP [18]. The mechanism that may lead to increased BP in people with high hemoglobin levels is not fully understood; however, several biological mechanisms have been proposed for the association between hemoglobin and BP [18–20].

HTN is called a silent disease, since it does not have obvious clinical symptoms. Identifying the factors associated with HTN can be effective in better identifying and controlling the disease as well as predicting the prognosis of patients [21, 22]. Given the importance of HTN and its short- and long-term complications and the existence of contradictory results in studies on the relationship between BP and hemoglobin, this study aims to investigate the cross-sectional association between hemoglobin concentration and BP in people aged 35–70 years in a cohort study of Rafsanjan, Iran.

## 2. Methods

### 2.1. Study Design

The present cross-sectional study was conducted based on Rafsanjan adult cohort study [23] as a part of the prospective epidemiological research studies in Iran (PERSIAN) [24]. In this cohort study, 10,000 people aged 35–70 years were selected from urban and rural areas, under the auspices of four health centers No. 1, 3, 4, and 8 using the list of population health records. The inclusion criteria in the cohort study included Iranian citizenship, an age range of 35–70 years, and residence for at least 9 months per year in the study area. The noninclusion criterion was severe physical and mental disorders. At the beginning of this study, informed consent was obtained from all the subjects to participate in the study. In this study, the standard PERSIAN Cohort study questionnaires consisting of 482 questions in 3 major sections of general, medical, and nutrition were completed by a trained interviewer. The validity and reliability of all questionnaires were confirmed in previous studies and prepared as a software program [24, 25]. After obtaining informed consent, face-to-face interviews were conducted by the interviewers, and their responses were collected electronically and confidentially. The systolic and diastolic BP of the participants was measured using a Riester mercury sphygmomanometer and appropriately sized cuff according to the American College of Cardiology/American heart Association (ACC/AHA) recommends. Participants should be relaxed and seated with legs uncrossed and back and arm supported. If possible, the patient should be seated five minutes before the reading. All clothing covering the cuff location should be removed. Blood pressure was measured two times with a one-minute interval between them, and the average of the measurements was recorded. Blood pressure levels were recorded regardless of the use of antihypertensive medications. Blood pressure readings are expressed in millimeters of mercury (mm Hg) [23, 26]. Blood pressure measurements were performed on the same day in which blood samples were drawn. Anthropometric measurements including; height, waist, hip, and wrist circumferences (in cm) and weight (in kg), were measured based on the US National Institutes of Health protocols [23, 27]. These measurements were taken in the morning while the participants were still fasting since these characteristics have minimum bias at this time. Weight was measured using a Seca scale with minimal clothing and height was measured using a Seca wall-mounted height-measure scale without shoes. Waist circumference (WC) and hip circumference (HC) were measured with a tape measure with minimal clothing in centimeter. Two trained nurse performed all measurements. Experimental biochemical indices were measured using BT 1500 (Chemistry Analyzer BT-1500 Biotecnica Instruments, Italy), and Pars Azmoon kits [28].

### 2.2. Participants

In the present study, all the participants in the Rafsanjan adult cohort study were examined. Exclusion criteria were people with incomplete information, a history of myocardial infarction, and those with very low hemoglobin concentrations (<11 mg/dl) [29]. Demographic and background information, including age, gender, marital status, education level, place of residence, employment status, race, smoking and alcohol use, pulse rate (beats per minute), weight, height, body mass index (BMI), physical activity, history of diabetes, HTN, ischemic heart disease and CVD (cardiovascular disease) in the individual, systolic and diastolic blood pressure, fasting blood sugar (FBS), triglycerides, cholesterol, low-density lipoprotein (LDL), high-density lipoprotein (HDL), blood urea nitrogen (BUN), creatinine (Cr), white blood cell (WBC), red blood cell (RBC), metabolic equivalent of task (MET), serum glutamic-oxaloacetic transaminase (SGOT), serum glutamic pyruvic transaminase (SGPT), alkaline phosphatase (ALP), gamma-glutamyl transferase (GGT), and hemoglobin concentration were extracted from the data available in Rafsanjan adult cohort study. Physical activity is defined by MET based on the scoring of the International Physical Activity Questionnaire (IPAQ) [29].

### 2.3. Statistical Analysis

Results are presented as mean ± SD (standard deviation) or median (1st quartile–3rd quartile) for numeric variables and are summarized by absolute frequencies and percentages for categorical variables. Numeric variables were compared using independent two-sample *t*-test or nonparametric Mann–Whitney *U* test, for skewed distributions, and categorical variables were compared using chi-square test across the gender spectrum.

Logistic regression analyses were conducted to assess the cross-sectional associations between hemoglobin concentrations at baseline and hypertension. For these analyses, we used 4 models including model 1: was an unadjusted model; model 2: was adjusted for age, gender, and body mass index; model 3: was adjusted for age, gender, body mass index, alcohol intake, smoking, cholesterol, LDL, HDL-C, MET final, BUN, and FBS; and model 4: was adjusted for age, gender, body mass index, alcohol intake, smoking, cholesterol, LDL, HDL-C, MET final, BUN, FBS, diabetes, and history of CVD.

For the statistical analysis, the statistical software SPSS version 24.0 for Windows (IBM SPSS Inc., Chicago, IL, USA) and SAS software version 9.2 (SAS Inc., Cary, NC) were used. All *p* values were 2-tailed, with statistical significance defined by *p* ≤ 0.05.

## 3. Results

In the present study, the information of 9398 participants (out of the total initial sample size of 10,000) was included in the final analysis (61 people due to incomplete information, 245 people due to hemoglobin less than 11 and 296 people due to myocardial infarction were excluded). Moreover, 4396 of the participants were male (46.8%) and 5002 were female (53.2%). Among women, 2485 (49.7%) were between 35 and 49 years (reproductive age).  Table 1 reveals the baseline demographic characteristics of the participants. The mean age of participants was 49.78 ± 9.53 years, and there was no significant age difference between males and females (*p*=0.953).

Based on the results of Table 2 in all cases, the difference between the studied variables between men and women was statistically significant (*p* < 0.05), except for the history of CVD (*p*=0.073). None of the women were on hormone therapy.

Table 3 represents the results of four models of logistic regression analysis to evaluate the cross-sectional association between hemoglobin concentration and BP. These results show that in males, no significant relationship was observed between BP and hemoglobin concentration in adjusted models 2 and 3. In females, unlike males, a positive and significant relationship was observed between BP and hemoglobin concentration in the studied models. In models 1, 3, and 4, the results indicate a higher odds ratio between BP and hemoglobin concentration higher than 13.7 g/dl. By increasing every unit of hemoglobin concentration in the blood in women, the odds ratio of rising BP increased from a minimum of 1.116 (95% CI: 1.035–1.203) in model 2 to a maximum of 1.185 (95% CI: 1.094–1.283) in model 4. This relationship was established in all the studied models.

The results of the general analysis of the population in examining the odds ratio of HTN and the first to fourth quartiles of hemoglobin show that in adjusted models 3 and 4, a positive and significant relationship was observed between BP and hemoglobin concentration. There was a significant relationship between the adjusted model 3 (OR = 1.062, 95% CI: 1.005–1.121) and the adjusted model 4 (OR = 1.090, 95% CI: 1.031–1.153).

**Figure 1:**
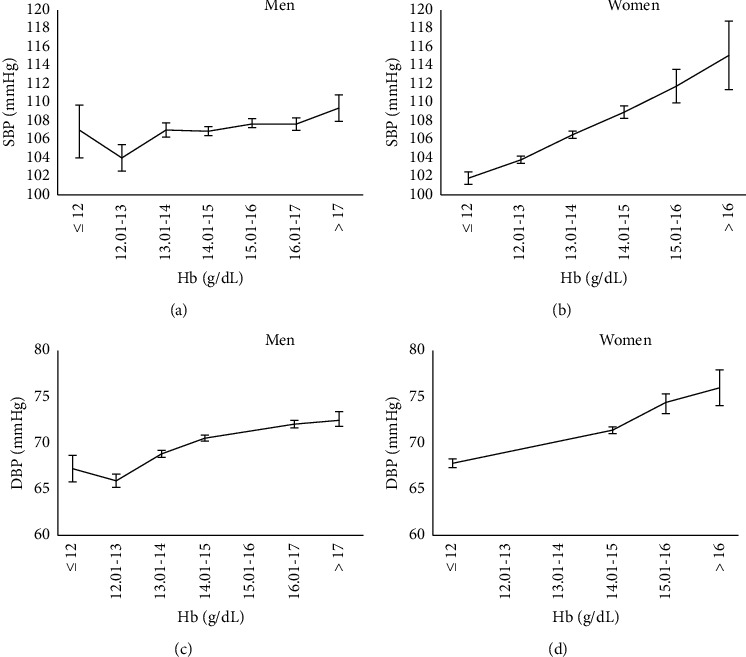
Trends of BP following the increase of hemoglobin concentrations at baseline (a) systolic BP in males, (b) systolic BP in females, (c) diastolic BP in males, and (d) diastolic BP in females.

Figure 1 reveals the trend between systolic and diastolic BP and hemoglobin concentration levels for males and females. There was a positive trend and a linear increase between BP and hemoglobin levels. It is worth mentioning that the slope of the increasing trend was higher in females than males.

## 4. Discussion

High BP is the most important risk factor for the development of CVD and a growing problem in the global community [30]. Although HTN is a preventable disease, its high prevalence worldwide and serious and dangerous complications for the organs in the body have made this disease a major health problem in all communities [7]. Identifying the factors associated with HTN can be effective in better diagnosis and control of the disease, as well as predicting the prognosis of patients [22]. In the present study, a positive association was observed between BP and hemoglobin concentration, and the odds ratio of rising BP was more in higher levels of hemoglobin.

The results of the present study reveal that, in general, the odds ratio of rising BP increases by high level of hemoglobin. In this regard, Xuan et al. reported that the level of hemoglobin has a positive correlation with systolic (*r* = 0.075, *p* < 0.001) and diastolic (*r* = 0.272, *p* < 0.001) BP. This relationship was not affected by age, BMI, serum creatinine, and LDL in both genders [18]. In the study by Inrig et al., mean score of diastolic BP was higher in participants with higher hemoglobin, but this association with systolic BP was not significantly different. Although increasing the doses of erythropoiesis-stimulating agent (ESA) and hemoglobin were significantly associated with a linear increase in diastolic blood pressure, this association was not consistently observed with an increase in systolic BP [30]. A study by Kim et al. in South Korea reported that the mean hemoglobin level in people with HTN was significantly higher than in people without HTN. The odds ratio of HTN by increasing hemoglobin concentration, after adjusting for the variables of age, gender, BMI, mild renal dysfunction, lifestyle, and disease history, was also observed. In the longitudinal analysis, the relative risk of HTN by increasing hemoglobin concentration before adjustion was 1.09 and after adjustion for the mentioned variables was 0.91. Finally, these findings showed that hemoglobin alone does not cause HTN [31]. The results of the study by Lee et al. showed that by increasing hemoglobin concentration, systolic and diastolic BP in males increased by 2.6 and 3.2 mm Hg, respectively. In multiple logistic regression analysis, hemoglobin concentration showed a positive and significant relationship with HTN, independent of other confounding variables in males and females [32]. The study by Atsma et al. on blood donors in the Netherlands showed that hemoglobin levels were positively correlated with systolic and diastolic blood pressure. BP regression coefficients for each unit increase in hemoglobin level in males and females were 1.3 and 1.8 mm Hg, respectively [17]. Gobel et al. reported a significant correlation between moderate arterial and systolic and diastolic BP and the number of red blood cells, hematocrit, and hemoglobin concentration. Mean arterial BP was higher in males than females and this association was along with higher values for red blood cell count, hemoglobin concentration, and hematocrit in males compared to females [33]. Although the processes that can increase BP associated with increased hemoglobin levels are still unclear, researchers have suggested that various mechanisms, including increased blood concentration along with increased hematocrit and hemoglobin [20, 34], changes in vascular resistance and vasoconstriction [19], and stress [35], are among the factors in examining the relationship between hemoglobin and blood pressure.

In the present study, the results of examining the relationship between BP and hemoglobin in males and females showed a positive trend and a linear increase between BP and hemoglobin levels, so that the increasing trend was more in females compared to males. In this regard, the results of Jamshidi-Naeini's study revealed that the amount of hemoglobin is positively associated to blood pressure, so that by increasing each unit of hemoglobin, systolic and diastolic BP increases by 0.57 and 0.72 mm Hg, respectively [36]. The significant correlation between BP and hematocrit, which is one of the important factors in blood viscosity, indicates the role of biological factors in the long-term control of blood pressure. It seems that the gender difference in BP may be due to stimulated red blood cells in males compared to females [34]. Significant changes in growth pattern, lifestyle, eating habits, and behavior are likely to affect hemoglobin levels in both genders. Hemoglobin concentration is an important diagnostic indicator for a person's health, which does not differ significantly between males and females in the prepubertal period, and differences appear only after the onset of menstruation [37, 38]. Another factor affecting gender-related differences in hemoglobin concentration may be the direct effect of estrogens and androgens sex hormones on erythropoiesis [38], in the present study the trend of BP was higher by increasing hemoglobin in women. It can be indicated that due to the fact that blood concentrations in males are usually higher, their response is better by increasing hemoglobin levels. However, in females, there is less adaptation and response to the increase in hemoglobin level, resulting in higher blood pressure. In future studies, this issue should be further investigated, and if these findings are confirmed, its mechanisms should be assessed.

The present study has strengths and limitations. The use of large sample sizes, data collection, and the availability of different indicators for each individual made it possible to examine the relationship between BP and hemoglobin with greater accuracy. Moreover, logistic regression analysis and 4 models of analysis to evaluate the relationship between hemoglobin concentration and blood pressure, made it possible to control confounding variables. Antihypertensive drugs were not included in the statistical model, which was another limitation of this study. On the other hand, in this study, there was no information about the use of hormone replacement therapy (HRT) in postmenopausal women or iron supplements. Due to the nature of the study, it was not possible to examine the precedence and delay of whether high hemoglobin is more associated with BP or vice versa; therefore, it needs to be evaluated in prospective studies.

## 5. Conclusion

The results of the study showed that the odds ratio of rising BP was more in higher levels of hemoglobin. There was also a positive trend and a linear increase between BP and hemoglobin levels. Given that determining the factors associated with HTN and developing HTN is valuable in identifying and controlling the disease and predicting the prognosis of patients, people with higher hemoglobin levels can be considered at risk.

## Figures and Tables

**Table 1:** Baseline demographic characteristics of the study participants based on gender.

Variables	Total (*n* = 9398)	Men (*n* = 4396)	Women (*n* = 5002)	Test statistics	*p* values
Age (year)	49.78 ± 9.53	49.78 ± 9.70	49.79 ± 9.39	*t* = −0.059	0.953^**#**^
Employment status				*χ* ^2^ = 5547.808	<0.001^*∗*^
Jobless	147 (1.6)	129 (2.9)	18 (0.4)		
Employed	4502 (47.9)	3543 (80.6)	959 (19.2)		
Retired	978 (10.4)	724 (16.5)	254 (5.1)		
Housewife	3771 (40.1)	0	3771 (75.4)		
Education level				*χ* ^2^ = 199.202	<0.001^*∗*^
Illiterate	879 (9.4)	269 (6.1)	610 (12.2)		
≤Diploma	6975 (74.2)	3204 (72.9)	3771 (75.4)		
Academic	1544 (16.4)	923 (21.0)	621 (12.4)		
Marital status				*χ* ^2^ = 421.224	<0.001^*∗*^
Single	125 (1.3)	59 (1.3)	66 (1.3)		
Married	8668 (92.2)	4293 (97.7)	4375 (87.5)		
Widowed	493 (5.2)	17 (0.4)	476 (9.5)		
Divorced	112 (1.2)	27 (0.6)	85 (1.7)		
Current smoking	1592 (16.9)	1515 (34.5)	77 (1.5)	*χ* ^2^ = 1802.537	<0.001^*∗*^
Drug use	2199 (23.4)	2002 (45.5)	197 (3.9)	*χ* ^2^ = 2259.374	<0.001^*∗*^
Alcohol intake	964 (10.3)	953 (21.7)	11 (0.2)	*χ* ^2^ = 1170.417	<0.001^*∗*^

Data are expressed as mean ± standard deviation (SD) or number (%). ^*∗*^Chi-square test; ^#^Independent two-sample *t*-test.

**Table 2:** Baseline clinical and anthropometry characteristics of the study participants based on gender.

Variables	Total (*n* = 9398)	Men (*n* = 4396)	Women (*n* = 5002)	Test statistics	*p* values
Weight (kg)	73.63 ± 13.43	76.03 ± 14.01	71.52 ± 12.52	*t* = 16.360	<0.001^**#**^
Height (cm)	162.86 ± 9.36	170.37 ± 6.58	156.26 ± 5.79	*t* = 109.644	<0.001^**#**^
Waist circumference (cm)	95.86 ± 11.52	92.50 ± 11.26	98.81 ± 10.92	*t* = −27.536	<0.001^**#**^
Hip circumference (cm)	101.33 ± 9.23	98.19 ± 7.38	104.10 ± 9.80	*t* = −33.242	<0.001^**#**^
BMI (kg/m^2^)	27.81 ± 4.83	26.14 ± 4.28	29.28 ± 4.80	*t* = −33.465	<0.001^**#**^
MET	37.68 (35.27–40.40)	37.58 (34.70–43.21)	37.75 (35.75–39.63)	*Z* = −3.368	0.001^†^
Systolic BP (mmHg)	106.22 ± 16.94	107.25 ± 16.49	105.32 ± 17.28	*t* = 5.537	<0.001^**#**^
Diastolic BP (mmHg)	70.68 ± 10.23	71.37 ± 10.32	70.07 ± 10.11	*t* = 6.147	<0.001^**#**^
Pulse rate (beats per minute)	74.23 ± 9.54	71.52 ± 8.96	76.62 ± 9.39	*t* = −26.875	<0.001^**#**^
RBC (M/*µ*L)	5.16 ± 0.52	5.45 ± 0.49	4.90 ± 0.41	*t* = 58.639	<0.001^**#**^
WBC (K/*µ*L)	6.50 (5.50–7.60)	6.60 (5.60–7.70)	6.40 (5.40–7.40)	*Z* = −8.007	<0.001^†^
Hemoglobin (g/dL)	14.07 ± 1.39	15.04 ± 1.16	13.22 ± 0.94	*t* = 82.918	<0.001^**#**^
Hematocrit (%)	42.742 ± 3.99	45.51 ± 3.35	40.31 ± 2.73	*t* = 81.772	<0.001^**#**^
Cholesterol (mg/dl)	199.42 ± 37.45	195.34 ± 36.53	203.00 ± 37.88	*t* = −9.981	<0.001^**#**^
HDL (mg/dl)	57.93 ± 10.82	54.41 ± 9.46	61.02 ± 10.99	*t* = −31.320	<0.001^**#**^
LDL (mg/dl)	108.82 ± 29.76	106.63 ± 28.89	110.74 ± 30.38	*t* = −6.710	<0.001^**#**^
FBS (mg/dl)	101.00 (94.00–112.00)	101.00 (94.25–111.00)	102.00 (94.00–114.00)	*Z* = −2.754	0.006^†^
BUN (mg/dl)	14.00 (11.00–16.00)	14.00 (12.00–17.00)	13.00 (11.00–15.00)	*Z* = −23.015	<0.001^†^
Creatinine (mg/dl)	1.00 (0.90–1.10)	1.10 (1.00–1.20)	0.90 (0.90–1.00)	*Z* = −59.752	<0.001^†^
Triglyceride (mg/dl)	145.00 (106.00–199.00)	151.00 (109.00–212.00)	141.00 (103.00–191.00)	*Z* = −8.295	<0.001^†^
SGOT (U/L)	18.00 (15.00–22.00)	19.00 (16.00–23.00)	17.00 (14.00–20.00)	*Z* = −23.472	<0.001^†^
SGPT (U/L)	18.00 (13.00–25.00)	20.00 (15.00–30.00)	16.00 (12.00–22.00)	*Z* = −22.920	<0.001^†^
ALP (U/L)	216.00 (181.00–260.00)	219.00 (187.00–259.00)	212.00 (176.00–260.00)	*Z* = −5.289	<0.001^†^
GGT (U/L)	22.00 (16.00–30.00)	25.00 (19.00–35.00)	19.00 (15.00–26.00)	*Z* = −26.623	<0.001^†^
Diabetes	1774 (18.9)	634 (14.4)	1140 (22.8)	*χ * ^2^ = 107.008	<0.001^*∗*^
HTN	2069 (22.0)	635 (14.4)	1434 (28.7)	*χ * ^2^ = 275.708	<0.001^*∗*^
Ischemic heart disease	615 (6.5)	268 (6.1)	347 (6.9)	*χ * ^2^ = 2.704	0.100^*∗*^
History of CVD	714 (7.6)	311 (7.1)	403 (8.1)	*χ * ^2^ = 3.215	0.073^*∗*^

Data are expressed as mean ± standard deviation (SD), median (1^st^ quartile–3^rd^ quartile), or number (%). RBC: red blood cell; HDL: high-density lipoprotein-cholesterol; LDL: low-density lipoprotein; MET: metabolic equivalent of task; WBC: white blood cell; FBS: fast blood sugar; BUN: blood urea nitrogen; SGOT: serum glutamic-oxaloacetic transaminase.

**Table 3:** Cross-sectional association between hemoglobin concentrations and HTN at baseline.

Baseline hemoglobin concentration (g/dL)	No. of people	Odds ratio (95% confidence interval) for rising BP			
		Model 1^*∗*^	Model 2^†^	Model 3^‡^	Model 4^#^
Men					
<14.3	977	1.00	1.00	1.00	1.00
14.3–14.9	1013	0.850 (0.669–1.080)	1.006 (0.777–1.303)	1.063 (0.817–1.383)	1.096 (0.837–1.434)
15.0–15.5	979	0.777 (0.608–0.993)	0.926 (0.710–1.208)	1.004 (0.766–1.316)	1.072 (0.813–1.413)
≥15.6	1427	0.691 (0.550–0.868)	0.801 (0.625–1.026)	0.869 (0.674–1.120)	0.939 (0.725–1.217)
Continuous (per 1 g/dL)	4396	0.895 (0.833–0.963)	0.921 (0.852–0.995)	0.946 (0.874–1.024)	0.971 (0.895–1.053)
Women					
<12.6	1175	1.00	1.00	1.00	1.00
12.6–13.0	964	0.988 (0.811–1.205)	0.897 (0.716–1.124)	0.901 (0.715–1.135)	0.904 (0.714–1.146)
13.1–13.6	1306	1.176 (0.982–1.407)	0.912 (0.742–1.120)	0.935 (0.757–1.155)	0.961 (0.775–1.192)
≥13.7	1557	1.665 (1.407–1.971)	1.152 (0.949–1.398)	1.229 (1.006–1.501)	1.302 (1.061–1.596)
Continuous (per 1 g/dL)	5002	1.311 (1.227–1.400)	1.116 (1.035–1.203)	1.151 (1.064–1.244)	1.185 (1.094–1.283)
Total^₤^					
*Q*1	2573	1.00	1.00	1.00	1.00
*Q*2	2322	1.077 (0.946–1.226)	0.974 (0.841–1.128)	1.013 (0.872–1.177)	1.045 (0.897–1.217)
*Q*3	2309	0.870 (0.761–0.994)	1.202 (1.017–1.421)	1.289 (1.086–1.529)	1.363 (1.145–1.623)
*Q*4	2194	0.546 (0.471–0.633)	1.134 (0.922–1.394)	1.225 (0.990–1.515)	1.357 (1.093–1.685)
Continuous (per 1 g/dL)	9398	0.864 (0.834–0.896)	1.040 (0.986–1.096)	1.062 (1.005–1.121)	1.090 (1.031–1.153)

HTN: hypertension. ^*∗*^Model 1: unadjusted. ^†^Model 2: adjusted for age, gender, body mass index. ^‡^Model 3: adjusted for age, gender, body mass index, alcohol intake, smoking, cholesterol, LDL, HDL, MET, BUN, and FBS. ^#^Model 4: adjusted for age, gender, body mass index, alcohol intake, smoking, cholesterol, LDL, HDL, MET, BUN, FBS, history of diabetes, and CVD. ^₤^Quartiles of baseline hemoglobin concentration.

## Data Availability

The datasets generated and/or analyzed during the current study are not publicly available due to PERSIAN cohort policy on availability of health care registers but are available from the corresponding author on reasonable request.
